# Motor learning by selection in visual working memory

**DOI:** 10.1038/s41598-021-87572-6

**Published:** 2021-04-29

**Authors:** Ilja Wagner, Christian Wolf, Alexander C. Schütz

**Affiliations:** 1 AG Allgemeine und Biologische Psychologie, Philipps-Universität Marburg, Gutenbergstraße 18, 35039 Marburg, Germany; 2 Allgemeine Psychologie, Westfälische Wilhelms-Universität, Münster, Germany; 3 Center for Mind, Brain and Behavior, Marburg, Germany

**Keywords:** Human behaviour, Saccades, Working memory

## Abstract

Motor adaptation maintains movement accuracy over the lifetime. Saccadic eye movements have been used successfully to study the mechanisms and neural basis of adaptation. Using behaviorally irrelevant targets, it has been shown that saccade adaptation is driven by errors only in a brief temporal interval after movement completion. However, under natural conditions, eye movements are used to extract information from behaviorally relevant objects and to guide actions manipulating these objects. In this case, the action outcome often becomes apparent only long after movement completion, outside the supposed temporal window of error evaluation. Here, we show that saccade adaptation can be driven by error signals long after the movement when using behaviorally relevant targets. Adaptation occurred when a task-relevant target appeared two seconds after the saccade, or when a retro-cue indicated which of two targets, stored in visual working memory, was task-relevant. Our results emphasize the important role of visual working memory for optimal movement control.

## Introduction

Only the center of the human visual field, the fovea, allows high acuity vision. Consequently, humans use saccadic eye movements to reorient their fovea to relevant locations in the world. However, saccades are not always accurate in guiding gaze to targets: a variety of factors, such as pathologies^1^ or aging^2^ may cause inaccuracies in their execution. To maintain saccade accuracy throughout the lifespan^3^, these errors need to be compensated by motor adaptation.

In the laboratory, saccade adaptation is studied by displacing an otherwise irrelevant target during the saccade to induce a postsaccadic error^4^ (for reviews, see^5,6,7^). Targets are typically irrelevant to the participant, that is, they serve no other purpose than being the saccade target. Using variations of this paradigm, it has been shown that saccade adaptation is primarily driven by prediction errors, i.e., the difference between the actual and predicted target position after the saccade^8^. Similar to other cases of motor learning, the cerebellum is crucial for saccade adaptation^9^ (for a review, see^10^). Furthermore, it is thought that the cerebellum corrects saccade inaccuracies most effectively when it receives feedback about saccade accuracy in a time window between 0 and 300 ms after saccade termination. By disturbing this immediate feedback, for example by turning off the target during a saccade and only turning it back on after a delay of 400–1500 ms, saccade adaptation decreases with increasing delay and is essentially absent at the longest feedback-delays^11–13^.

Outside the laboratory, however, a myriad of stimuli simultaneously competes for limited processing resources and humans rarely spontaneously saccade to behaviorally irrelevant targets that do not possess any relevance other than that they can serve as eye movement targets. Instead, humans saccade to objects they consider interesting^14^ or aim to interact with^15^. A white cup in a kitchen, for example, will most likely not cause spontaneous visual exploration. However, asking observers to prepare some tea would make the same object relevant for the current behavioral task and promote it to an attractive target for saccades, which are necessary for visual exploration and guidance of further interactions with the object (e.g., grasping it). Unlike artificial laboratory settings, saccades in naturalistic scenarios do not necessarily yield feedback about saccade accuracy immediately after movement termination: a saccade to a tea cup, which has to be lifted from a shelf during tea preparation, is typically terminated several seconds before the cup is reached and grasped by the hand^16,17^. Consequently, the oculomotor system receives feedback about saccade quality (i.e., if it was suitable to guide the hand to the cup, in order to lift it safely) only several seconds after saccade termination^16^. The short postsaccadic time window of error evaluation reported in laboratory studies (up to about 400 to 1500 ms) would not be useful for this natural asynchrony of saccades, action execution and action consequences.

Therefore, to successfully optimize eye movement control without immediate postsaccadic feedback, the brain would need to be able to delay error evaluation for saccade adaptation until action consequences become apparent, long after saccade offset. To allow this, information about an eye movement target would need to be stored for later usage. A potential resource, responsible for holding this kind of information, is visual working memory (VWM), which is known for its role in the storage and active maintenance of information about no longer visible stimuli^18–20^. Here we tested if visual working memory allows for flexible error evaluation in motor adaptation by asking human participants to saccade to an arrangement of two stimuli and memorize their orientation. Seconds after saccade termination, one memorized stimulus was cued as being relevant for a memory task: Through this, the brain received no meaningful information about saccade quality (i.e., whether the saccade accurately guided the fovea to the task-relevant stimulus) immediately after eye movement offset, but only after cue presentation, long after saccade termination. If error evaluation for saccade adaptation can be delayed to compensate the natural asynchrony between saccades, action execution and action consequences, we would expect participants to compute an error signal based on the memory representation of the task-relevant stimulus and to use this error signal to correct the erroneous saccade amplitude for the next time the same eye movement is executed.

## Results

### Trial-by-trial changes in saccade amplitude

**Figure 1:**
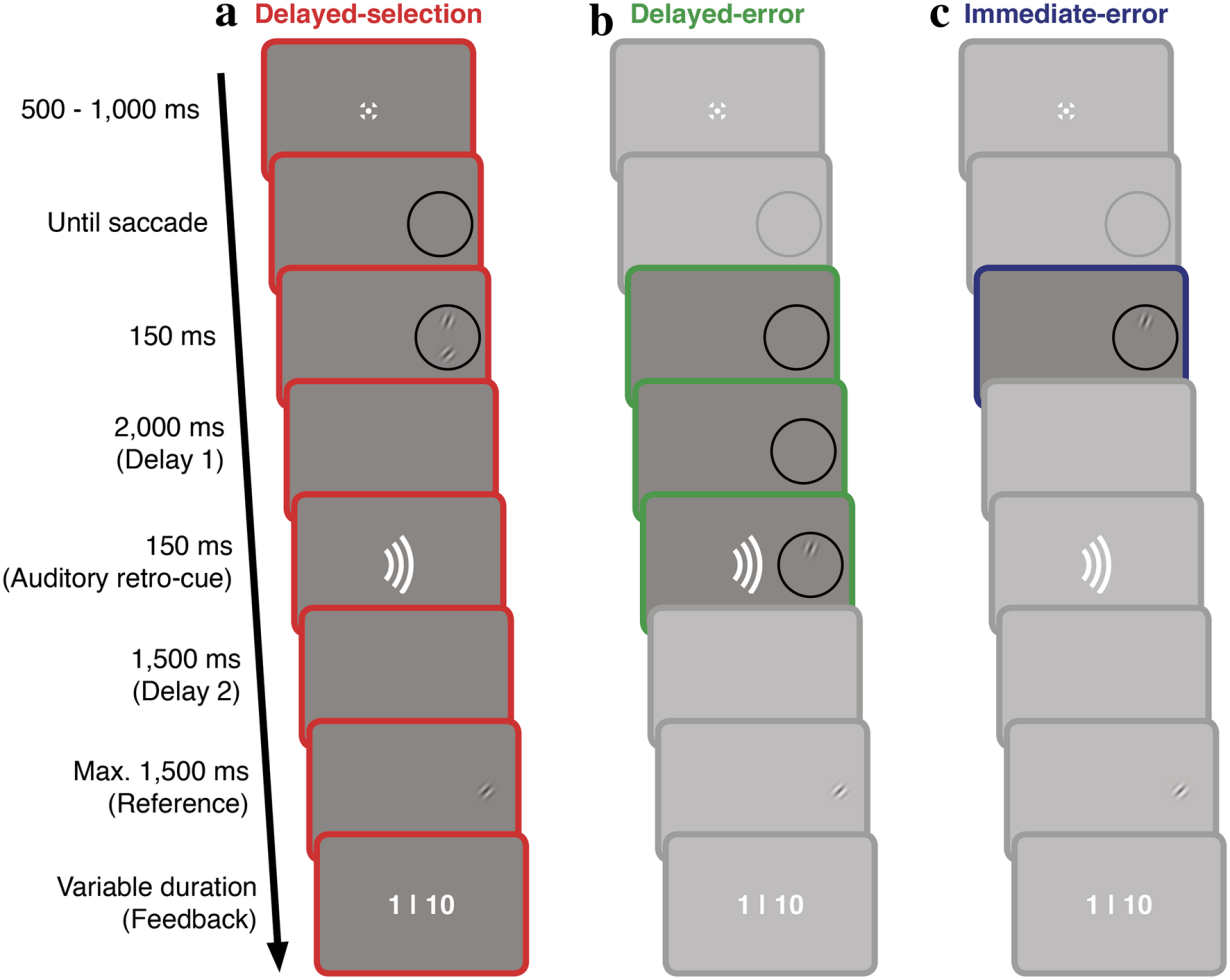
Trial procedure. (**a**) In the delayed-selection condition, participants were instructed to saccade to an otherwise irrelevant eye movement target (black ring), which always appeared 10° to the right of the fixation cross. During the saccade, two stimuli (Gabor patches) were shown within the eye movement target. After delay 1, one of the two stimuli was cued as being task-relevant by an auditory retro-cue. Thus, the task-relevant stimulus in this condition always corresponds to the one out of two shown stimuli, signaled by the retro-cue as being relevant for the comparison task of our paradigm. After delay 2, a reference (Gabor patch) was shown and participants had to judge if the orientation of the reference matched the orientation of the task-relevant (i.e., cued) stimulus. Participants received feedback about their judgement at the end of a trial. (**b**) In the delayed-error condition, only one task-relevant stimulus was shown after delay 1. (**c**) In the immediate-error condition, only one task-relevant stimulus was shown immediately after the saccade. (**b**,**c**) In the latter two conditions, since only one stimulus was shown before reference onset, this singular stimulus was automatically the task-relevant stimulus that had to be compared to the reference. Differences in the trial procedure relative to the delayed-selection condition are highlighted. (**a**–**c**) Participants were allowed to look around freely during delays and were not required to maintain fixation. The screen remained empty during both delays, except for the delayed-error condition, in which we showed the ring throughout delay 1. Stimuli are not drawn to scale.

To study the flexibility of error evaluation in motor adaptation, we combined a saccade adaptation paradigm with a visual working memory paradigm that involves a delayed-match to sample task (Fig. 1). Our paradigm consisted of three conditions (delayed-selection, delayed-error and immediate-error). In all conditions we asked participants, first, to execute a saccade to an eye movement target (a black ring) and, second, to compare a subsequently presented task-relevant stimulus to a reference presented at the end of the trial. The task-relevant stimulus appeared within the eye movement target, but vertically off with regard to its center. This induced a postsaccadic error and eventually saccade adaptation. Due to the presence of a memory-task in all three conditions of this experiment, we call this the memory-task experiment.

In the delayed-selection condition of the memory-task experiment, two vertically displaced stimuli were shown immediately after the saccade and only after a 2.15 s delay (delay 1), an auditory retro-cue indicated which stimulus was task-relevant and had to be compared to the reference, shown after another 1.5 s delay (delay 2). Thus, in this condition, we define the task-relevant stimulus as the one out of the two shown stimuli which was marked by the retro-cue as relevant for the comparison to the reference. For saccade adaptation to occur in this condition, error evaluation would require memorizing the saccade endpoint and both stimulus positions until auditory retro-cue presentation. The delayed-selection condition, therefore, constitutes our main condition and tests if information, stored in VWM, can be used to compute error signals for motor adaptation. In the delayed-error condition, only one task-relevant stimulus appeared after delay 1: this condition constitutes our first control condition, included to estimate the temporal decay of adaptation with delayed error evaluation as in previous studies^11–13^. In the immediate-error condition, one task-relevant stimulus was shown immediately after the saccade: this constitutes our second control condition, included to estimate the maximal adaptation effect with immediate, unambiguous postsaccadic error evaluation. In delayed-error and immediate-error trials, participants saw one stimulus before reference onset, making this stimulus task-relevant for the comparison to the reference; the retro-cue in the latter two conditions was uninformative and, in contrast to the delayed-selection condition, the stimulus location does not need to be maintained in VWM to compute an error signal for saccade adaptation. We used the established random-step procedure^21,22^ for all conditions, such that the cued stimulus (delayed-selection) or the stimulus location (delayed-error and immediate-error) were random over trials, making them unpredictable for participants.

**Figure 2:**
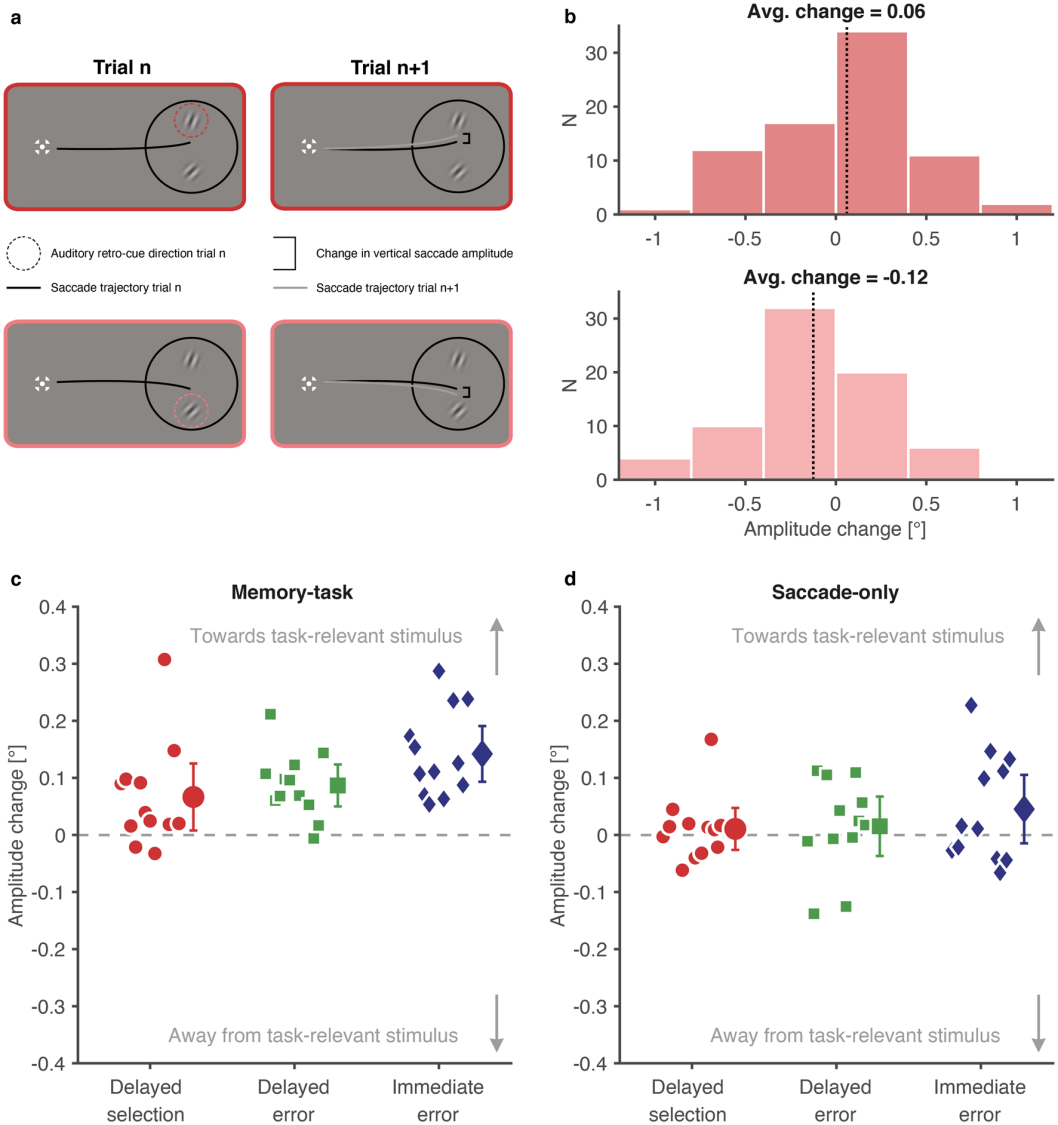
Amplitude changes in the direction of the task-relevant stimulus. (**a**) Illustration of the expected changes in the vertical saccade amplitude from trial n (left column) to trial n + 1 (right column), when the upper (first row) or lower (second row) stimulus was cued as being task-relevant in trial n. The shown saccade trajectories do not represent actual data. (**b**) Distributions of trial-to-trial amplitude changes when the upper (first row) or lower (second row) stimulus was cued as being task-relevant. The displayed data stems from one representative participant. Vertical saccade amplitudes should become more positive/negative in trial n + 1 when the upper/lower stimulus was cued as being task-relevant in trial n. (**c**,**d**) Trial-to-trial changes in the vertical saccade amplitude in the memory-task (**c**) and saccade-only experiment (**d**). Although participants in the saccade-only experiment were not involved in the memory task, we still randomly presented one of two auditory retro-cues in each trial. (**c**,**d**) Small symbols denote individual participant’s data, large symbols the respective group mean. Error bars represent 95% confidence intervals.

Participants in all conditions of the memory-task experiment performed the delayed-match to sample task above chance (proportion correct delayed-selection: *M* = 0.75, *CI*_*95%*_ [0.67, 0.82]; delayed-error: 0.88, *CI*_*95%*_ [0.83, 0.93], immediate-error: 0.81, *CI*_*95%*_ [0.73, 0.89]). To determine if error evaluation for saccade adaptation can be delayed until saccade quality becomes apparent, we analyzed trial-to-trial changes in vertical saccade amplitude (Fig. 2a,b). If the position of one out of two stimuli held in VWM can be used to calculate an error signal for saccade adaptation in a given trial (n), we should observe that the amplitude in the subsequent trial (n + 1) changes towards the stimulus cued in trial n. In accordance with this prediction, we found systematic trial-to-trial amplitude changes towards the position of the cued stimulus in the delayed-selection condition (*M* = 0.07°, *CI*_*95%*_ [0.01°, 0.13°]) (Fig. 2c). This was also true in the delayed-error condition, where only one task-relevant stimulus was shown seconds after saccade offset (*M* = 0.09°, *CI*_*95%*_ [0.05°, 0.12°]). Compared to the delayed-selection and delayed-error condition, the effect in the immediate-error condition, in which one task-relevant stimulus was shown immediately after saccade termination, was slightly larger (*M* = 0.14°, *CI*_*95%*_ [0.09°, 0.19°]). Effects were consistent in all conditions: the majority of participants showed mean trial-to-trial changes in the cued direction (delayed-selection: 10/12, delayed-error: 11/12, immediate-error 12/12) and the magnitude of the observed amplitude changes (3.5–7.0% of the target error) corresponds to typical magnitudes of single-trial learning (0.5–24%)^21–24^. Moreover, the distributions of amplitude changes for the two stimulus positions were sufficiently distinct to allow predicting the stimulus location in trial n based on the observed amplitude changes from trial n to n + 1 (Supplementary Fig. S1, Supplementary Information).

Earlier studies, investigating how saccade adaptation evaluates error signals^11–13^, reported that saccade adaptation relies on immediate feedback after saccade termination. However, despite introducing a 2.15 s delay between saccade termination and presentation of the task-relevant stimulus, we still found saccade amplitude changes in our delayed-error condition (Fig. 2c). One possible explanation for the discrepant result might be that our memory task temporally bound saccade and task-relevant stimulus together, which might not hold true for task-irrelevant targets, used in earlier studies. Therefore, if task-relevance was the driving factor behind delayed error evaluation for saccade adaptation, then making stimuli task-irrelevant should yield similar results to earlier studies^11–13^. To test this hypothesis, we repeated our paradigm with a new sample of participants. This new sample performed the same three conditions as in the memory-task experiment, but participants were only instructed to make a saccade to the eye movement target and were no longer instructed to judge the orientation of the stimuli (see Methods for a detailed description). Due to the absence of a memory task, we call this second experiment the saccade-only experiment.

Without memory task, trial-to-trial changes in vertical saccade amplitude were considerably weaker (Fig. 2d). This was true for the delayed-selection (*M* = 0.01°, *CI*_*95%*_ [− 0.03°, 0.05°]), delayed-error (*M* = 0.02°, *CI*_*95%*_ [− 0.04°, 0.07°]) and immediate-error condition (*M* = 0.05°, *CI*_*95%*_ [− 0.01°, 0.11°]). Furthermore, the observed effects were less consistent compared to the memory-task experiment (delayed-selection: 7/12 participants showed mean trial-to-trial changes in the cue direction, delayed-error: 7/12 participants, immediate-error: 7/12 participants). A linear mixed model with fixed effects of condition (delayed-selection, delayed-error, immediate-error) and experiment (memory-task, saccade-only) showed main effects of experiment, *F*(1, 22) = 15.05, *p* < 0.001, and condition, *F*(2, 44) = 3.43, *p* = 0.041, but no interaction between those factors, *F*(2, 44) = 0.44, *p* = 0.650.

To summarize, trial-to-trial changes in saccade amplitude occurred when one out of two task-relevant stimuli, held in visual working memory, was cued long after saccade offset (delayed-selection) or when a single task-relevant stimulus was presented long after saccade offset (delayed-error). Furthermore, the observed amplitude changes were moderated by the presence of the memory task; without the latter, participants showed generally weaker changes.

### Gaze bias

So far, we found consistent trial-to-trial amplitude changes towards the position of the task-relevant stimulus in the previous trial. This effect could be based on two entirely different mechanisms. First, it could reflect adaptation of the sensorimotor transformation, in the sense that saccade targeting remains unchanged and only the resulting motor plan is adapted. This would suggest that motor adaptation can be driven by signals arriving long after movement termination. Second, amplitude changes could reflect a lingering attentional bias, in the sense that saccade targeting is not only determined by the signal from the peripheral saccade target but also by lingering activity from the previous trial. Such an attentional bias might arise from memorizing the task-relevant stimulus and its location for the subsequent response and should also be apparent in a consistent bias in gaze position over the course of one trial^25,26^.

To dissociate these hypotheses, we inspected the average gaze position between primary saccade onset in trial n and 700 ms after reference onset in trial n + 1. If the observed trial-to-trial amplitude changes reflect adaptation of sensorimotor transformation, then we expect a gaze bias towards the location of the task-relevant stimulus in the same trial (trial n). This gaze bias should disappear after the information held in VWM becomes obsolete^25,26^. However, if amplitude changes reflect a lingering attentional bias, we expect the gaze bias from trial n to still be present in trial n + 1.

**Figure 3:**
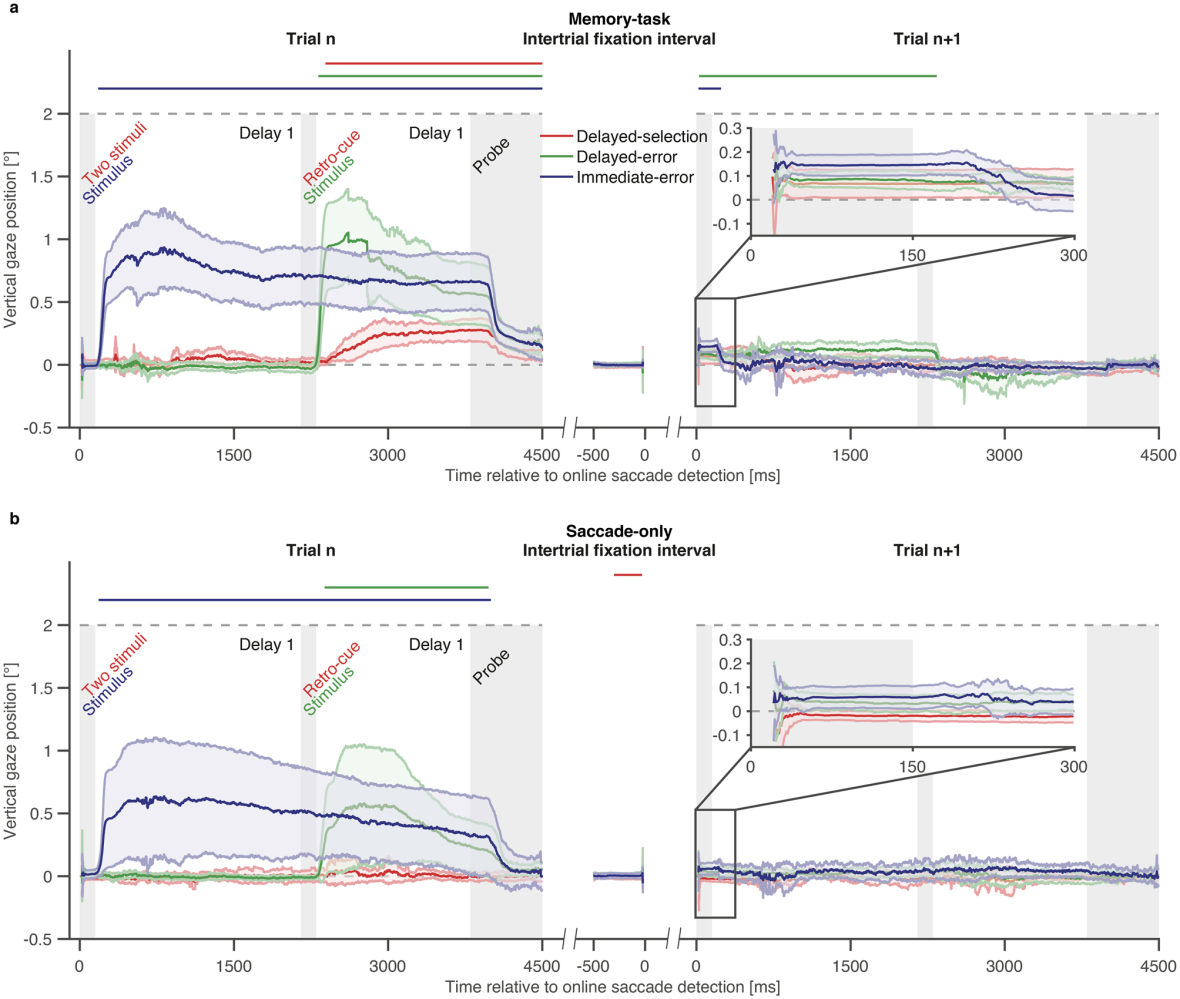
Gaze bias in trial n and n + 1. (**a**) The three colored lines represent the average vertical gaze position as a function of time in the memory-task experiment; positive gaze positions represent gaze positions closer to the location of the task-relevant stimulus in trial n. Colored shaded areas represent 95% confidence intervals. Colored horizontal bars above each plot mark significant clusters in the respective condition. The position of the task-relevant (+ 2°) stimulus in trial n is indicated by the dashed, grey line. In trial n + 1, the task-relevant stimulus appeared randomly at a vertical position of + 2° or − 2°. Grey shaded areas mark, from left to right, display duration of the stimulus (in the delayed-selection and immediate-error condition), the presentation duration of the auditory retro-cue (and display duration of the task-relevant stimulus in the delayed-error condition), and the first 700 ms of the response phase. The two areas between the grey shaded areas represent delay 1 and 2, respectively. The data are shown time-locked to online saccade detection. (**b**) Average vertical gaze position as a function of time in the saccade-only experiment. Conventions are the same as in panel (**a**). (**a**,**b**) Insets in trial n + 1 represent the zoomed in data from the first 300 ms after online saccade detection.

To test our hypotheses, we performed a cluster-based permutation analysis. This analysis showed that participants in the delayed-selection condition of the memory-task experiment developed a gaze bias towards the location of the task-relevant stimulus during delay 2, Σ*T* (sum of *t*-values of the largest cluster) = 11,335.29, *p* < 0.001. The gaze bias started 241 ms after retro-cue onset and declined back to screen center 2109 ms later, shortly after reference onset (Fig. 3a). Most critically, the gaze bias was absent during the last 500 ms before online saccade detection (i.e., the initial fixation interval) in the subsequent trial (n + 1), excluding the possibility that the observed trial-to-trial amplitude changes simply reflect a lingering attentional bias. The absence of a gaze bias during the end of the fixation interval cannot be explained by the presence of the fixation cross alone: previous work, in which participants memorized a task-relevant stimulus, found a gaze bias even in the presence of a fixation cross throughout the trial^25,26^. Moreover, the gaze bias did not reappear after the primary saccade in trial n + 1.

Participants also showed systematic modulations of their average gaze position in trial n of the delayed-error condition, Σ*T* =  11,889.10, *p* < 0.001, 171–2350 ms after stimulus onset, and immediate-error condition, Σ*T* = 28,157.61, *p* < 0.001, 179–4500 ms after stimulus onset. Those modulations temporally coincided with the presentation of the task-relevant stimulus and were accompanied by additional reactive or corrective saccades towards the task-relevant stimulus (Supplementary Fig. S2, Supplementary Fig. S3, Supplementary Information). Those larger saccades were mostly absent in the delayed-selection condition, where the observed gaze bias was primarily driven by small (micro)saccades, as in previous reports of this phenomenon^25^. As in the delayed-selection condition, we found no gaze bias during the fixation interval in trial n + 1 of the delayed-error and immediate-error condition. However, a gaze bias emerged during delay 1 of trial n + 1 in the delayed-error, Σ*T* =  11,007.13, *p* < 0.001, 28–2340 ms ms after ring onset, and immediate-error condition, Σ*T* =  1475.89, *p* =  0.010, 21–241 ms after stimulus onset, but disappeared shortly after offset of the respective task-relevant stimulus. The rise of the gaze bias in trial n + 1 simply reflects the trial-to-trial amplitude changes (Fig. 2c). Since there was no visual stimulation after the saccade in the delayed-error condition, the gaze bias persisted until the next task-relevant stimulus appeared. Indeed, the decline of the gaze bias in trial n + 1 of the delayed-error and immediate-error condition temporarily coincided with the presentation of the respective task-relevant stimulus. The location of task-relevant stimuli in two consecutive trials was uncorrelated. Thus, in half of the trials the presentation of the task-relevant stimulus in trial n + 1 might have favored a gaze bias and reactive/corrective saccades to the upper part of the screen, whereas it was the other way for the remaining half.

Without memory task there was no gaze bias in the delayed-selection condition (Fig. 3b, Supplementary Fig. S4). In the delayed-error condition, a gaze bias emerged during delay 2, Σ*T* =  4259.71, *p* = 0.003, 231–1827 ms after stimulus onset. In the immediate-error condition, a gaze bias was present during delays 1 and 2, Σ*T* =  11,110.68, *p* =  0.006, 184–3999 ms after stimulus onset. Here, the onset of the gaze bias temporarily coincided with the presentation of the task-relevant stimulus and went along with additional reactive or corrective saccades (Supplementary Fig. S4) towards the task-relevant stimulus. We found no gaze bias in trial n + 1 of the delayed-error and immediate-error condition. In the delayed-selection condition, a gaze bias was present during the fixation interval in trial n + 1, Σ*T* = − 714.48, *p* =  0.021,  − 301–29ms before online saccade detection.

To summarize, we demonstrated that revealing the task-relevant stimulus in all three conditions of our memory-task experiment induced a systematic gaze bias towards the location of the task-relevant stimulus. This gaze bias even developed without additional visual stimulation (delayed-selection), persisted until the end of one trial and, in the following trial, only reemerged in the delayed-error condition, when the task-relevant stimulus was not apparent immediately. We interpret the absence of any gaze bias during the fixation interval in all conditions as evidence that trial-to-trial amplitude changes (Fig. 2c) were most likely the consequence of adapting the corresponding motor plan, rather than a lingering attentional bias influencing saccade targeting in trial n + 1.

## Discussion

Previous studies on eye movement adaptation showed that the motor system is sensitive to errors only within a very brief temporal interval of up to about 400 to 1500 ms after the movement^11–13^. Here, we found trial-by-trial amplitude changes in saccades related to a task-relevant stimulus that either appeared or was selected in visual working memory (VWM), long after saccade termination. This suggests that the motor system is more flexible than previously thought and that it can evaluate errors in VWM well after the end of the movement.

These results have important implications for three distinct lines of state-of-the-art research that we will expend in the following paragraphs: First, our results clearly emphasize the need for more natural paradigms when studying motor control and learning. Second, our findings establish a new functional role of visual working memory in supporting motor learning, and highlight that the content of visual working memory can bias action control^27^ (for reviews, see^28–30^). Third, our results and experimental paradigm are of direct relevance for the neurophysiology of motor learning.

Studies on visuomotor adaptation of limb movements also emphasize the need for more naturalistic paradigms when studying motor learning. Classical studies showed that adaptation of reaching movements in prism adaptation^31–34^ and visuomotor rotation paradigms^35^ is reduced with increasing delay of feedback, similarly to saccade adaptation. However, visuomotor adaptation is not only sensitive to errors at the end of the movement, but also at the end of the task^36^. It is obvious that learning from task errors is an indispensable ability to optimize any action with temporally delayed outcome, such as a basketball throw or a golf swing. However, considering that humans make multiple saccades per second, one might question the ecological validity of our paradigm and the importance of evaluating error signals of saccades, executed seconds ago, and potentially followed by subsequent eye movements. Additionally, the primary goal of saccade adaptation is to maintain accurate saccades over a lifespan^3^: a task which, theoretically, could be achieved by just taking into account immediate saccade consequences (did they bring the fovea close enough to the saccade target?), without waiting for delayed information about action outcome.

However, as outlined in the introduction, saccades are not only crucial for extracting information for cognitive processing, as in reading, but they are also necessary to guide interactions with one’s environment^16^ (for a review, see^37^). Although considering only immediate saccades consequences might be sufficient for simple exploration tasks, delayed consequences might be crucial when dealing with more complex behaviors, such as eye-hand coordination. Similarly, although humans can execute multiple saccades per second, the pace is much slower in eye-hand coordination^16^. Our results of delayed error-attribution and -evaluation are also in line with the recent observation that motor learning can be elicited even in the absence of movements^38^.

Past research has provided evidence that saccade targets are automatically stored in VWM^39,40^ (for a review, see^41^) and that gaze behavior can be influenced by the contents of VWM and vice versa (for reviews, see^28,29^). Sensory processing used for saccade programming can be modulated by VWM contents: eye movements are initiated more quickly and executed more accurately if the visual target matches a target held in VWM^42^, landing position is biased towards the matching target if multiple targets compete^43^ and matching VWM content can speed up visual search for postsaccadic targets^44^. It is unlikely that such modulations of sensory processing, induced by VWM contents, occurred in our paradigm because the eye movement target (ring) was distinct from the stimulus held in visual working memory (Gabor patch). However, two recent studies^45,46^ reported that residual activity from memorizing a target for memory-guided saccades in one trial can persist until the following trial, systematically biasing memory-guided saccade endpoints there. Although those effects bear some qualitative resemblance to trial-to-trial amplitude changes from our study (Fig. 2c), we are convinced that our results cannot be explained by lingering memory activity from trial n − 1 because of two reasons: First, participants in our paradigm made visually, not memory-guided saccades. Papadimitriou et al.^45^, however, report that the influence of persisting memory activity vanished when their participants had to make visually guided saccades. Second, Papadimitriou et al.^45^ report that saccade endpoints in memory-guided saccade trials were still biased to the target location from the previous trial, when the latter contained visually guided saccades, implying that actively memorizing a target’s location is not necessary to bias saccade endpoints. This would predict that merely seeing a stimulus in trial n should systematically bias saccade endpoints in trial n + 1 towards the previous stimulus location even in the delayed-error and immediate-error condition of our saccade-only experiment. However, the observed effects in that experiment were close to zero (Fig. 2d). Although those arguments indicate that our results cannot (at least not fully) be attributed the lingering memory activity, they cannot fully rule-out this alternative interpretation and additional experiments would be required.

Finally, we can ask how saccade adaptation with delayed errors might be accomplished on a neural level. Adaptation of saccades with immediate errors has been shown to rely on the cerebellum (for reviews, see^10,47,48^). The Marr-Albus theory of cerebellar learning^49,50^ postulates that motor errors induce complex spikes (CS) in Purkinje cells, which modulate simple spike activity, eventually causing a reduction of the experienced motor error^51^. The error signal, communicating an erroneous movement and inducing modulations in CS activity, most likely originates in the superior colliculus (SC): stimulating the SC after a saccade induces adaptation^52^ and blocking the error signal from the superior colliculus reduces adaptation^53^. A previous study reported that error signals induce error-related activity in CS within a critical error interval, ~ 80–120 ms after termination of inaccurate eye movements and before execution of any corrective eye movements^52^.

Participants in our delayed-selection condition, however, did not receive feedback about eye movement accuracy until two seconds after movement offset. One way, in which saccade adaptation might still occur under such conditions, would be that the SC generates multiple error signals in the critical error interval and relays those signals to the cerebellum for later evaluation. Each of those error signals would indicate an inaccurate eye movement relative to one of our two displayed stimuli. Instead of immediately translating the incoming error signals into motor adaptation, however, the cerebellum might delay error evaluation in one of two ways: it could either store both error signals and later select and process the signal relative to the task-relevant stimulus. Alternatively, the cerebellum could process both error signals immediately after their arrival and just translate the relevant correction into behavior, after the cue indicates the location of the task-relevant stimulus.

In the last years, a growing body of literature demonstrated that the cerebellum is not only involved in motor control and coordination, but also that it contributes to cognitive functions, such as VWM and visual attention (for a review, see^54^). One study^55^, for example, demonstrated that lobule VIIb and VIIIa of the cerebellum encode visual field representations, similar to spatial maps found in the cerebral cortical dorsal attention network. The latter are theorized to constitute attentional priority maps that represent prioritized portions of the visual space^56,57^. Those maps might enable the cerebellum to temporarily store, a), the location of previously seen stimuli, and b), multiple incoming error signals, the latter are then tied to specific stimulus representations, before the sum of both signals is translated into behavior. The selection of the appropriate error signal/correction might take place through a shift of attentional resources towards the relevant representation within the cerebellar map^58^.

In addition to its pivotal role in learning from motor errors, the cerebellum has been shown to be involved in reinforcement learning (for a review, see^59^). For instance, Purkinje cells represent a reinforcement-learning signal during the acquisition of a sensorimotor association task^60^. Such reinforcement-learning signals might be the physiological basis of motor adaptation driven by reinforcement^61,62^ and a way how eye movements can be optimized for behavioral outcomes of whole eye-hand coordination sequences.

The previous remarks on the neural basis of delayed motor learning, as observed in our paradigm, assume that saccade adaptation to task-relevant stimuli is based on the same core mechanism as saccade adaptation elicited by intra-saccadic target steps^4^. So far, there are only a few behavioral studies on saccade adaptation to task-relevant stimuli^23,24,63^ (for a review, see^6^). Although some recent studies pointed out that, besides the cerebellum, higher cortical areas might contribute to saccade adaptation in general^64,65^, there is no physiological data specifically on task-driven saccade adaptation. Hence, saccade adaptation in our paradigm might have been caused by an entirely different neural mechanism than traditional saccade adaptation. In this case, a different underlying mechanism might be the explanation for the longer temporal sensitivity profile to error signals.

## Methods

### Participants

We recorded 13 participants for the memory-task experiment and 19 participants for the saccade-only experiment, similar to previous studies on saccade adaptation. Participants with less than 45% of valid trials (i.e., trials that were not excluded from analysis, see Eye movement and data analysis) in at least one condition were excluded from the analyses. This applied to one participant in the memory-task experiment and five participants in the saccade-only experiment. Two additional participants (saccade-only) were excluded because they voluntarily opted out after the first session. In the saccade-only experiment, participants 19 (delayed-selection and delayed-error condition), 11 (immediate-error condition) and 15 (delayed-error condition) had to repeat the first block of the respective measurement because of problems during data collection. The remaining 12 participants in the memory-task experiment had a mean age of 24 years (range: 20–30, eight female). The remaining 12 participants in the saccade-only experiment had a mean age of 23 years (range: 20–28, 9 female).

All participants provided informed consent prior to testing and were naïve to the purpose of the experiments. All experiments were conducted in accordance with the ethical guidelines laid down in the 1964 declaration of Helsinki and were approved by the ethics committee of the Marburg University, Department of Psychology (proposal 2017-27 k). All participants had normal or corrected-to-normal vision as well as unimpaired color vision. Participants either received course credit or were compensated with 8 €/h.

### Equipment

All experiments were conducted using the Psychtoolbox^66^ in MATLAB R2016b (The MathWorks, Natick, MA, USA). Stimuli were presented on a back-projection setup, using a PROPixx projector (VPixx Technologies Inc., Saint-Bruno, Quebec, Canada) and a Stewart Filmscreen screen (Stewart Filmscreen Corporation, Torrance, California, USA). The screen had a size of 90.7 × 51.0 cm, a spatial resolution of 1920 × 1080 pixel and a refresh rate of 120 Hz. The viewing distance was 106 cm. Background luminance was 70 cd/m^2^ and the screen was calibrated to ensure a linear gamma correction. A hotspot correction was used to ensure equal luminance across the screen. Eye movements of the right eye were recorded with an EyeLink 1000 + (SR Research Ltd., Ontario, Canada), at a sampling rate of 1000 Hz. The Eyelink Toolbox was used to control the eye tracker^67^. Auditory stimuli were presented on Sennheiser HD 280 Pro headphones (Sennheiser Electronic GmbH and Co. KG, Wedemark, Germany), which participants wore throughout all measurements.

### Stimuli

A combination of cross and bull’s eye (diameter: 0.6°) was used as fixation cross^68^. Gabor patches were used as stimuli in all conditions. The underlying sine-wave grating had a spatial frequency of 1.5 cycles per degree (cpd) and a Michelson contrast of 0.6. Gratings were seen through a Gaussian window with a standard deviation of 0.4°.

In each trial of the delayed-selection condition, both stimuli were displayed with different orientations (possible orientations: 22.5°, 45°, 67.5°, 112.5°, 135°, 157.5°). To ensure that the stimuli in a given trial were never displayed with the same orientation, a distance of 90° between their two respective orientations was used. The reference either had the same orientation as the stimulus that was cued as being task-relevant, or it was tilted by ± 22.5° relative to the task-relevant stimulus’ orientation. Therefore, although the reference could have the same orientation as the cued stimulus, it never had the same orientation as the non-cued stimulus. In the immediate-error and delayed-error condition, the same stimuli were used; here, however, only one Gabor patch was shown in a given trial. In all three conditions, a black ring with a radius of 4° and a line thickness of 0.05° served as the eye movement target.

### Design

The memory-task experiment consisted of three conditions: delayed-selection, delayed-error and immediate-error condition. Additionally, one control experiment, the saccade-only experiment, was recorded with a separate group of participants. The saccade-only experiment was mostly identical to the memory-task experiment, with the major difference being that participants did not have to perform a memory task (see Procedure and Instruction of participants).

A within-participant design was used for both experiments. Each participant attended four sessions and completed one condition per session. The first session was a training condition to familiarize participants with the memory task. In sessions 2–4 participants completed one of the experimental conditions (i.e., condition was manipulated across sessions) with the order being counterbalanced across participants. Sessions took place on different days and individual sessions were separated by at least one day.

### Instruction of participants

Participants in both experiments were instructed to, first, look at a central fixation cross, and second, to make a saccade to the eye movement target (the black ring) as soon as it appeared in the periphery. In the memory-task experiment, participants were additionally instructed to judge the orientation of a subsequently presented stripped pattern. This additional instruction was omitted for participants in the saccade-only experiment. In both experiments, participants received no instruction about how to behave during the delays, allowing them to look around freely. Participants were instructed to look back to the screen center after reference offset, where feedback was presented.

The instruction for the memory-task experiment included additional information about the memory task (which stimulus is cued by which auditory retro-cue, which keys to use for response, how to interpret the feedback screen and under which circumstances participants gained/lost points). The saccade-only instruction referred to the feedback message as “a random sequence of numbers”, instead of “feedback”.

### Procedure

#### Training condition

The training condition consisted of 96 trials. At the start of each trial a fixation cross was shown at the screen center. After a uniform random time-interval between 0.5 and 1 s, the eye movement target, an empty ring, appeared 10° to the right of the fixation cross. Participants were instructed to saccade towards the ring, as soon as it appeared. After saccade detection, and while the saccade was in flight, a Gabor patch was displayed at the center of the ring. The Gabor patch and the ring were removed from the screen 150 ms after saccade offset. If no saccade was detected 500 ms after onset of the eye movement target, the empty ring was removed.

After stimulus removal, a 2 s delay (delay 1) was presented, during which participants saw a grey, but otherwise empty screen. Afterwards, an uninformative auditory retro-cue was presented: The cue was either high-pitched (${f}_{high}=250$ Hz) or low-pitched (${f}_{low}=150$ Hz) and had a duration of 150 ms. After cue presentation, another delay (delay 2) was presented, which lasted 1.5 s. After the second delay, a Gabor patch (the reference) appeared at the former center of the ring and participants were instructed to judge by button press if the reference had the same orientation as the previously seen Gabor patch. Participants were given 1.5 s for their response.

After judging the stimulus orientation, participants received visual feedback about their trial performance. The feedback contained the score for the current trial as well as the overall score (for example “+ 1 | 42”). Participants received one point if they judged the orientation of the reference correctly and lost a point for an incorrect response. Irrespective of their response, participants did not receive a point if the executed saccade was erroneous (see Eye movement and data analysis) or if they missed to judge the references orientation within the given timeframe. In the latter case, the feedback at the end of a trial was displayed in red instead of black. For errors in eye movement behavior, a message was displayed instead of feedback, informing participants about their error. To achieve a constant intertrial interval, the display duration of the feedback was variable. It had a minimum value (500 ms) to which up to three values could be added: the difference (1) between the maximum and actual random fixation interval, (2) between the maximum and actual display duration of the peripheral target and (3) between maximum and actual response time. Through this, the time between two consecutive presentations of the fixation cross was constant.

#### Delayed-selection condition

The delayed-selection condition consisted of 200 trials. Two breaks were included after trials 67 and 134, respectively. During the breaks, participants were allowed to stand up and leave the room. After each break, the eye tracker was recalibrated. The trial procedure of the delayed-selection condition was identical to the training condition in terms of timing and feedback but differed with regard to the shown stimuli and the memory task. In the delayed-selection condition, two Gabor patches, vertically displaced by ± 2° from the ring center, were presented within the ring after saccade onset. Participants were informed that one of those two Gabor patches will be relevant for a subsequent memory task. However, which of the two Gabor patches was relevant was only revealed after delay 1, by playing either the high-pitched (cueing the upper stimulus as being relevant) or low-pitched auditory retro-cue (cueing the lower stimulus as being relevant). The cue direction was randomized across trials, which is based on established random-step designs, used previously to study saccade adaptation^21,22^. Before the task, participants performed 20 demonstration trials.

#### Immediate-error and delayed-error condition

The immediate-error and delayed-error condition each consisted of 200 trials and two breaks. Both conditions were identical to the delayed-selection condition in all regards, except the stimulus presentation. In the immediate-error condition, instead of two Gabor patches, one Gabor patch was shown within the ring, randomly appearing either at a position of + 2° or − 2°, vertically displaced from the screen center. Here, the auditory retro-cue always corresponded to the position of the Gabor patch (for example, a high-pitched cue was played when the Gabor patch appeared at a position of + 2°). Thus, participants in the immediate-error condition, as opposed to the delayed-selection condition, could evaluate saccade accuracy directly after eye movement offset.

Similarly, in the delayed-error condition only one Gabor patch was shown. However, it was not shown directly after saccade offset, but instead after delay 1, together with the auditory retro-cue. In this condition, the ring did not disappear after saccade offset, but stayed on the screen throughout delay 1. Ring and Gabor patch disappeared after the auditory retro-cue was played. Similar to the immediate-error condition, the auditory retro-cue in the delayed-error condition corresponded to the position of the Gabor patch.

#### Saccade-only experiment

The saccade-only experiment also contained three experimental and one training condition. All conditions were identical to the memory-task experiment with regard to trial number, procedure and stimuli. However, in the saccade-only experiment, participants did not have to solve a memory task. To rule out the possibility that the auditory retro-cue alone could drive trial-to-trial amplitude changes, we presented retro-cues, following the same logic as in the memory-task experiment. To match the experiments as much as possible in terms of timing and feedback, each participant from the saccade-only experiment was tested with the display duration of the reference (based on the mean response time of the matched participant) and the response feedback of a matching participant in the memory-task experiment. Matching participants were randomly assigned.

### Eye movement and data analysis

Saccade onsets and offsets were detected with the EyeLink algorithm. We only considered trials with a primary saccade latency between 80 and 400 ms, a horizontal amplitude between 7 and 13° and if gaze did not deviate more than 2° from the central fixation cross in a time window from − 20 to 80 ms relative to the eye movement target onset. Furthermore, we excluded trials with a blink during the primary saccade and trials without perceptual judgment (only memory-task experiment). Applying those criteria left, on average, 90.00% (delayed-selection condition), 89.79% (delayed-error) and 91.04% (immediate-error) valid (i.e., not excluded) trials in the memory-task experiment and 86.54% (delayed-selection), 89.29% (delayed-error) and 88.29% (immediate-error) valid trials in the saccade-only experiment. Valid trials were considered in all described analyses with the only exception being the analysis of trial-to-trial amplitude changes. Here, we additionally excluded all instances without valid eye movement data in two subsequent trials n and n + 1. Applying this additional criterion left, on average, 81.20% (delayed-selection), 81.16% (delayed-error) and 83.08% (immediate-error) valid trial trials in the memory-task experiment and 77.14% (delayed-selection), 80.78% (delayed-error) and 79.98% (immediate-error) valid trials in the saccade-only experiment. Trials with correct and incorrect perceptual judgments were analyzed jointly.

#### Gaze bias

To quantify gaze bias, we inspected the vertical component of the eye movement trace of each participant in a time window between primary saccade onset in trial n and 700 ms after reference onset in trial n + 1. Data points containing blinks and saccades were removed. Gaze traces in cue-down trials were recoded and analyzed together with traces from cue-up trials. Therefore, a positive gaze bias always indicates a gaze bias towards the cued stimulus, whereas a negative gaze bias indicates a gaze bias away from the cued stimulus.

#### Statistical analysis

We used linear mixed models for statistical inference. Mixed models were calculated using the nmle package^69^. Fixed effects of experiment and condition were categorically coded. Random effects were structured as random intercepts for each participant, except for analysis of interference by other saccades, where they were structured as random intercepts and slopes for condition and interval.

To reveal any gaze bias, we performed a cluster-based permutation test^70^ by using the implementation of Fieldtrip statistics^71^ in EEGLAB^72^. This method performs a two-sided *t*-test for each time point and clusters adjacent significant time points showing the same effect. For each cluster, the sum of *t*-values, Σ*T*, yields the cluster-level statistic. These original cluster-strengths were compared to a distribution of cluster-statistics that resulted from 10,000 permutations. For every permutation, data from the observed gaze bias traces and from a vector of zeros (i.e., baseline) were randomly permuted before the strongest cluster-level statistic was determined. The *p* value is given by 1 minus the percentile of the non-permuted cluster in the permuted distribution. Type-1 error rate for two-sided *t*-test was corrected by multiplying the *p* values of all found clusters by a factor of two.

## Supplementary Information

Supplementary Information

## Data Availability

Data is available from: https://doi.org/10.5281/zenodo.4618049
