# Supplementary material for: Motor learning by selection in visual working memory
Source: Sci Rep. 2021 Apr 29;11:9331. doi: 10.1038/s41598-021-87572-6 (PMC8085138; doi:10.1038/s41598-021-87572-6)
Supplement: Supplementary file 1 — Supplementary Information [file 41598_2021_87572_MOESM1_ESM.pdf]

Motor learning by selection in visual working memory

# – Supplementary Information –

Ilja Wagner, Christian Wolf & Alexander C. Schütz

Supplementary methods

Saccade amplitude distribution

We used the algorithm of Engbert & Kliegel [1] to offline detect small eye movements (microsaccades) within two time windows of interest: the time between primary saccade offset and auditory retro-cue onset as well as the time between auditory retro-cue onset and reference onset. Besides microsaccades, the algorithm also reliably detects regular saccades. To generate saccade amplitude distribution plots, we slid a moving window across the detected saccades and calculated how many saccades of a given amplitude we found per valid trial. The moving window had a width and step size of 0.01°. To account for postsaccadic fluctuations in saccade amplitudes, we only included saccades in the moving window, which were separated by at least 50 ms. Additionally, only saccades with amplitudes equal or greater than 0.10° were included. For each possible saccade amplitude, this analysis yielded a corresponding proportion of saccades with a given amplitude per trial. In a final step, the vector with those proportions was smoothed by a centered moving average with a window size of 0.10°.

Supplementary results

Prediction of the location of the task-relevant stimulus

Our main analysis showed substantial trial-to-trial amplitude changes in the vertical saccade amplitude, which systematically depended on the location of the task-relevant stimulus. However, this does not reveal if the distributions of amplitude changes for the two stimulus locations are sufficiently different to allow predicting the location of the task-relevant stimulus. Although averages are different, the distributions might be largely overlapping. To test this classification accuracy, we performed a receiver-operator characteristic (ROC) analysis and calculated the area under the ROC curve (AUC).

For this, the direction of the cue, presented in trial n, was used as predictor and the change in vertical saccade amplitude component between trial n and trial n+1 as criterion. The ROC analysis determines for each criterion (i.e., each observed trial-to-trial-change in saccade amplitude) with which rate it was caused by a cue of a given direction. The area under the resulting ROC curve serves as an index of discriminability between the distributions of trial-to-trial-changes in saccade amplitude after a high-pitched or a low-pitched auditory retro-cue was presented. If the distributions are identical, the AUC would yield a value of 0.5. If the presentation of a high-pitched/low-pitched auditory retro-cue in trial n goes along with a positive/negative change in saccade amplitude in trial n+1, the AUC would yield values > 0.5. AUC values < 0.5 indicate amplitude changes against the direction of the cue, presented in trial n (for example, the amplitude becomes more negative after an up cue was presented). Only trials with valid eye movement data in two subsequent trials (n and n+1) were included in the analysis (see Eye movement and data analysis in the main manuscript for trial exclusion criteria). We calculated the 95% confidence intervals across participants by using a bootstrapping procedure (10,000 samples). Therefore, the resulting confidence intervals might be asymmetric if the underlying data is not normally distributed.

The ROC analysis (Supplementary Fig. S1) revealed stronger and more consistent trial-to-trial amplitude changes towards the cued location in all conditions of the memory-task experiment (delayed-selection: $M = 0.57$, $CI_{95\%}$ [0.53, 0.64]; delayed-error: $M = 0.60$, $CI_{95\%}$ [0.56, 0.62]; immediate-error: $M = 0.65$, $CI_{95\%}$ [0.62, 0.70]), compared to the three conditions of the saccade-only experiment (delayed-selection: $M = 0.50$, $CI_{95\%}$ [0.48, 0.52]; delayed-error: $M = 0.52$, $CI_{95\%}$ [0.48, 0.56]; immediate-error: $M = 0.54$, $CI_{95\%}$ [0.50, 0.60]). A linear mixed model with fixed effects of condition (delayed-selection, delayed-error, immediate-error) and experiment (memory-task, saccade-only) yielded a main effects of condition, $F(2, 44) = 4.98$,

$p = 0.011$, and experiment, $F(1, 22) = 19.04$, $p < 0.001$, but no interaction, $F(2, 44) = 0.39$, $p = 0.677$.

Interference by other saccades during the delays

Since there was no visual target on the screen during the two delays, oculomotor behavior was unconstrained. The execution of other eye movements in that interval might interfere with adaptation, for instance when the delayed error feedback is not attributed to the primary saccade but to other saccades [2]. To investigate the interference by saccades during the delays, we focused our analysis on two time-windows of interest: the time between primary saccade offset and onset of the auditory retro-cue as well as the time between auditory retro-cue onset and onset of the reference. Within those windows, we detected (micro)saccades and analyzed their direction with respect to the task-relevant stimulus in the memory-task experiment.

In the delayed-selection condition of the memory-task experiment, participants on average directed an equal number of other saccades per trial towards ($M = 1.11$, $CI_{95\%}$ [0.51, 1.71]) and away from ($M = 1.11$, $CI_{95\%}$ [0.49, 1.73]) the location of the task-relevant stimulus after the primary saccade (Supplementary Fig. S2). After the cue, however, we found more saccades directed towards the location of the task-relevant stimulus ($M = 1.19$, $CI_{95\%}$ [0.75, 1.62]), compared to away from its location ($M = 0.88$, $CI_{95\%}$ [0.42, 1.34]) (Supplementary Fig. S2). In both time-windows of interest, saccades had relatively small amplitudes (Supplementary Fig. S2 & Supplementary Fig. S3), excluding the possibility that they simply re-allocated gaze to the memorized position of the no-longer-visible task-relevant stimulus [3]. This is similar to the previous finding of increased rates of small eye movements (microsaccades) towards the memorized location of a no longer visible stimulus [1].

We found a similar pattern in the delayed-error condition of the memory-task experiment: here, in-between primary saccade and target/cue-onset, (micro)saccades were directed equally often in both directions (Towards: $M = 1.23$, $CI_{95\%}$ [0.66, 1.80]; Away: $M = 1.28$, $CI_{95\%}$ [0.69, 1.88]) (Supplementary Fig. S2). After target/cue-onset however, more (micro)saccades were directed towards ($M = 1.50$, $CI_{95\%}$ [0.98, 2.02]) than away from the target location ($M = 1.10$, $CI_{95\%}$ [0.58, 1.62]) (Supplementary Fig. S2). In the immediate-error condition of the memory-task experiment the increase in the number of saccades towards the location of the task-relevant stimulus already occurred after the primary saccade (Towards: $M = 1.54$, $CI_{95\%}$ [1.07, 2.01]; Away: $M = 1.17$, $CI_{95\%}$ [0.61, 1.72]) (Supplementary Fig. S2),

whereas saccades towards and away from the location of the task-relevant stimulus occurred equally often after the cue (Towards: $M = 1.08$, $CI_{95\%}$ [0.66, 1.50]; Away: $M = 1.07$, $CI_{95\%}$ [0.66, 1.47]) (Supplementary Fig. S2). In contrast to the delayed-selection condition, we found substantially more saccades with larger amplitudes in the delayed-error and immediate-error condition (Supplementary Fig. S3). These larger saccades are most likely caused by the sudden appearance of the single task-relevant stimulus in those two conditions.

A linear mixed model with fixed effects of condition (delayed-selection, delayed-error, immediate-error), saccade direction (towards/away from the task-relevant stimulus) and interval (after primary saccade, after cue) showed a main effect of direction, $F(1, 66) = 46.10$, $p < 0.001$, but no main effects of interval, $F(1, 33) = 3.12$, $p = 0.087$, and condition, $F(2, 22) = 1.85$, $p = 0.180$. The three-way interaction between the factors was significant, $F(2, 66) = 23.69$, $p < 0.001$.

In the delayed-selection condition of the saccade-only experiment, we found no differential effect of the direction of other saccades, neither after the primary saccade (Towards: $M = 0.94$, $CI_{95\%}$ [0.65, 1.24]; Against: $M = 0.97$, $CI_{95\%}$ [0.67, 1.27]), nor after the cue (Towards: $M = 0.89$, $CI_{95\%}$ [0.63, 1.15]; Against: $M = 0.89$, $CI_{95\%}$ [0.67, 1.12]) (Supplementary Fig. S4). Similarly, the differential effect of other saccade direction was also absent in the delayed-error condition (Supplementary Fig. S4), both after the primary saccade (Towards: $M = 0.89$, $CI_{95\%}$ [0.46, 1.31]; Against: $M = 0.84$, $CI_{95\%}$ [0.42, 1.26]) as well as after the cue (Towards: $M = 1.17$, $CI_{95\%}$ [0.73, 1.60]; Against: $M = 1.11$, $CI_{95\%}$ [0.57, 1.64]). In the immediate-error condition (Supplementary Fig. 4), however, we found a differential effect after the primary saccade (Towards: $M = 1.47$, $CI_{95\%}$ [1.12, 1.82]; Against: $M = 1.22$, $CI_{95\%}$ [0.81, 1.62]), and a reversed effect after the cue (Towards: $M = 1.00$, $CI_{95\%}$ [0.64, 1.36]; Against: $M = 1.15$, $CI_{95\%}$ [0.73, 1.58]).

To summarize, we found that participants in all conditions of our two experiments executed saccades (and microsaccades) during the two delay periods. In the memory-task experiment the direction of other saccades was influenced by our auditory retro-cue as well as by the location of the task-relevant stimulus. We assume that saccades with small amplitudes most likely contributed to the development of the observed gaze bias, whereas saccades with large amplitudes most likely represent corrective and reactive saccades relative to the location of the visual stimuli. However, other saccades did not seem to interfere with adaptation of primary saccades in our paradigm: we found substantial trial-to-trial amplitude changes in the delayed-error and immediate-error condition of our memory-task experiment, whereas those

changes were mostly absent in the saccade-only experiment. This was the case, although participants executed a substantial number of other saccades in the respective conditions of both experiments. Most interestingly, despite a relatively high number of saccades during the delays, saccade adaptation in our paradigm successfully attributed an occurring motor error to the primary saccade, inducing systematic changes in this particular eye movement.

Supplementary figures

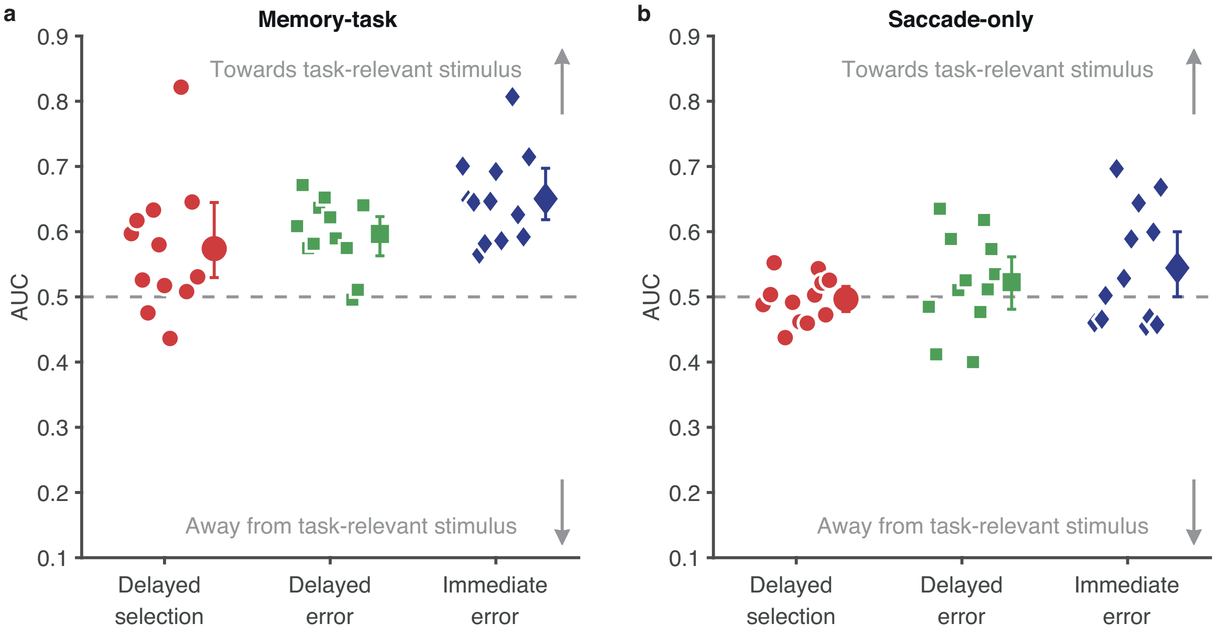

Supplementary figure S1. ROC analysis to predict the previous stimulus location based on amplitude changes. *Area under curve (AUC) values for the memory-task (a) and saccade-only (b) experiment. In all panels, small symbols denote individual data, large symbols the respective group mean. Error bars represent bootstrapped 95% confidence intervals.*

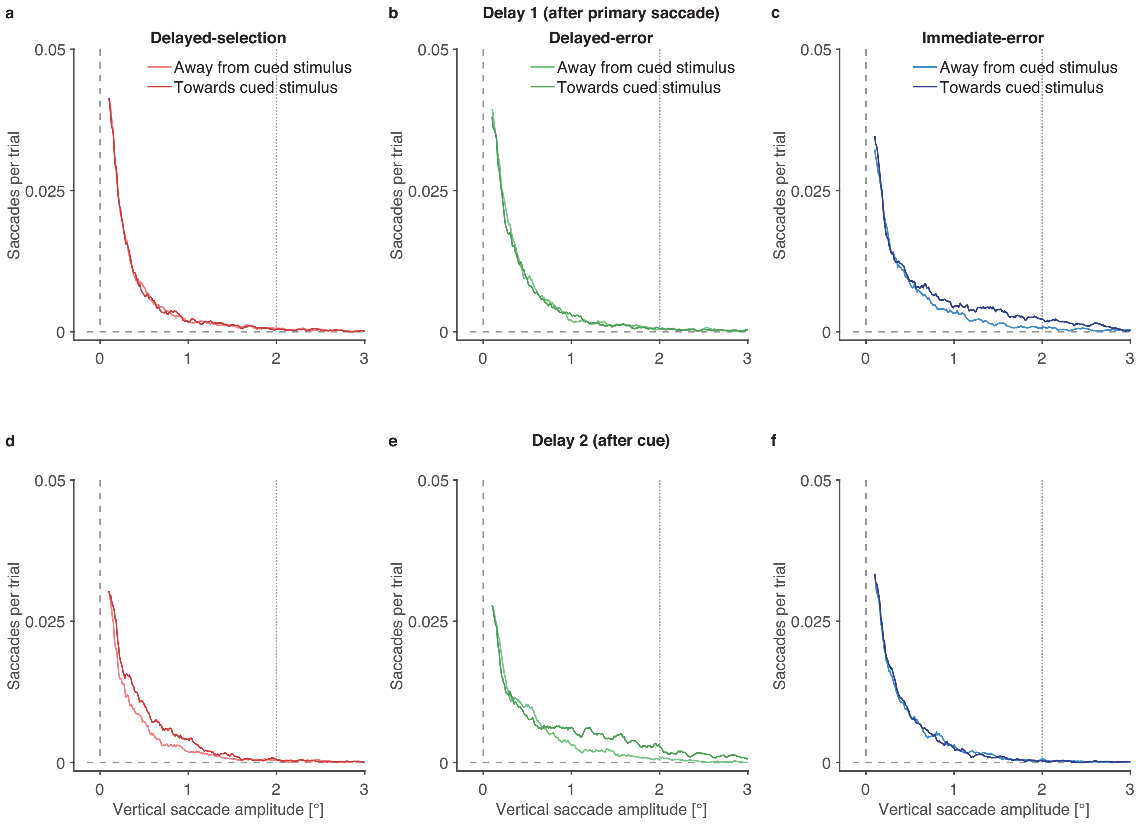

Supplementary figure S2. Saccade amplitude distribution during the delays in the memory-task experiment. *Distribution of vertical saccade amplitudes towards (saturated colors) and away (faint colors) from the location of the task-relevant stimulus in the three conditions of the memory-task experiment.* ***(a-c)*** *Interval between offset of the primary saccade and auditory retro-cue onset.* ***(d-f)*** *Interval between auditory retro-cue onset and reference onset. Solid colored lines indicate the number of saccades of a given amplitude, executed per trial. The grey dotted line marks the eccentricity of the stimuli.*

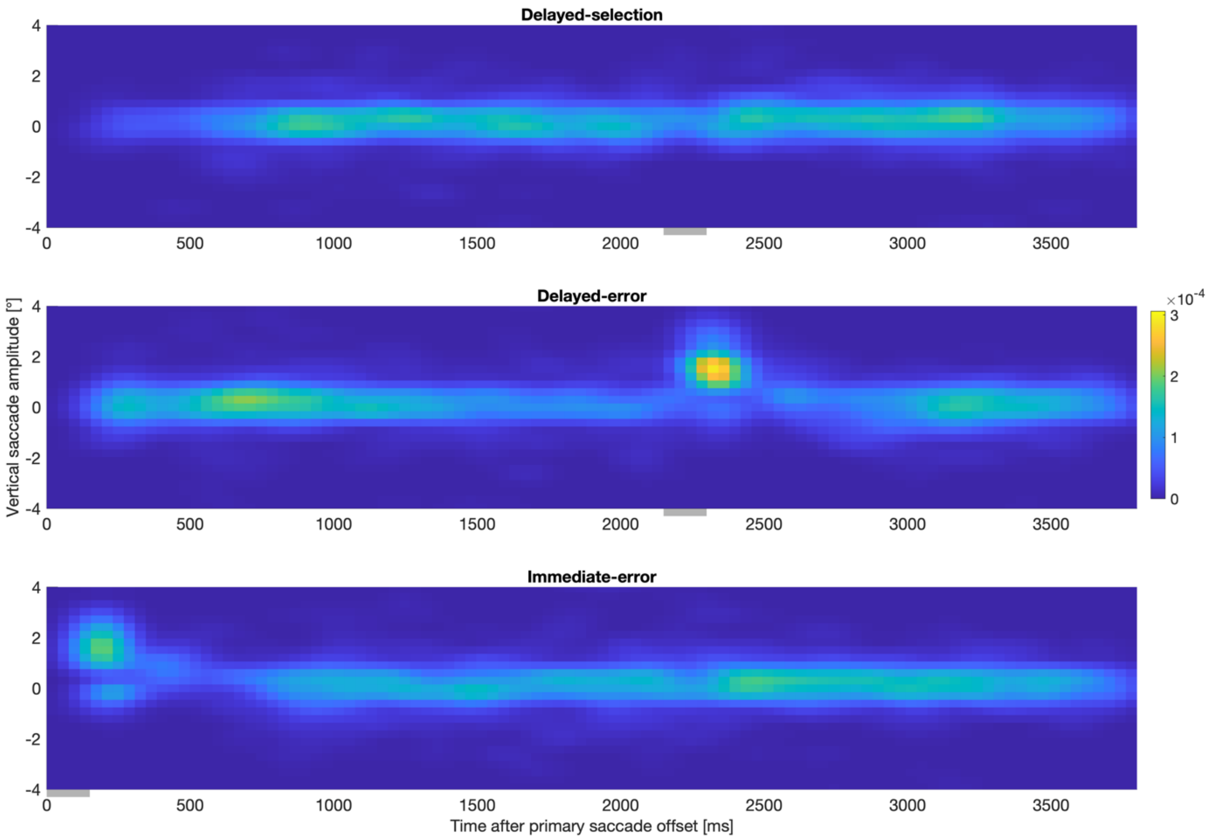

Supplementary figure S3. Probability density distributions for vertical saccade amplitudes in the two delay periods of the memory-task experiment. *Probability density estimates were computed by applying a kernel smoothing function on the bivariate data, corresponding to vertical saccade amplitudes and their onset time relative to primary saccade offset. The function evaluated combinations of 300 (x-axis) and 50 (y-axis) data-points, evenly spaced between the offset of the primary saccade and the onset of the reference (x-axis) as well as between -15° and 15° (y-axis). The kernel smoothing window had a bandwidth of 70 ms (x-axis) and 0.03° (y-axis). Amplitudes in cue-down trials were recoded, therefore, a positive amplitude always indicates a saccade towards the task-relevant stimulus, whereas a negative amplitude indicates a saccade away from it. Data is shown time-locked to primary saccade offset. Grey bars beneath the x-axis indicate time of auditory retro-cue presentation (delayed-selection) or presentation window of the task-relevant stimulus (delayed-error and immediate-error).*

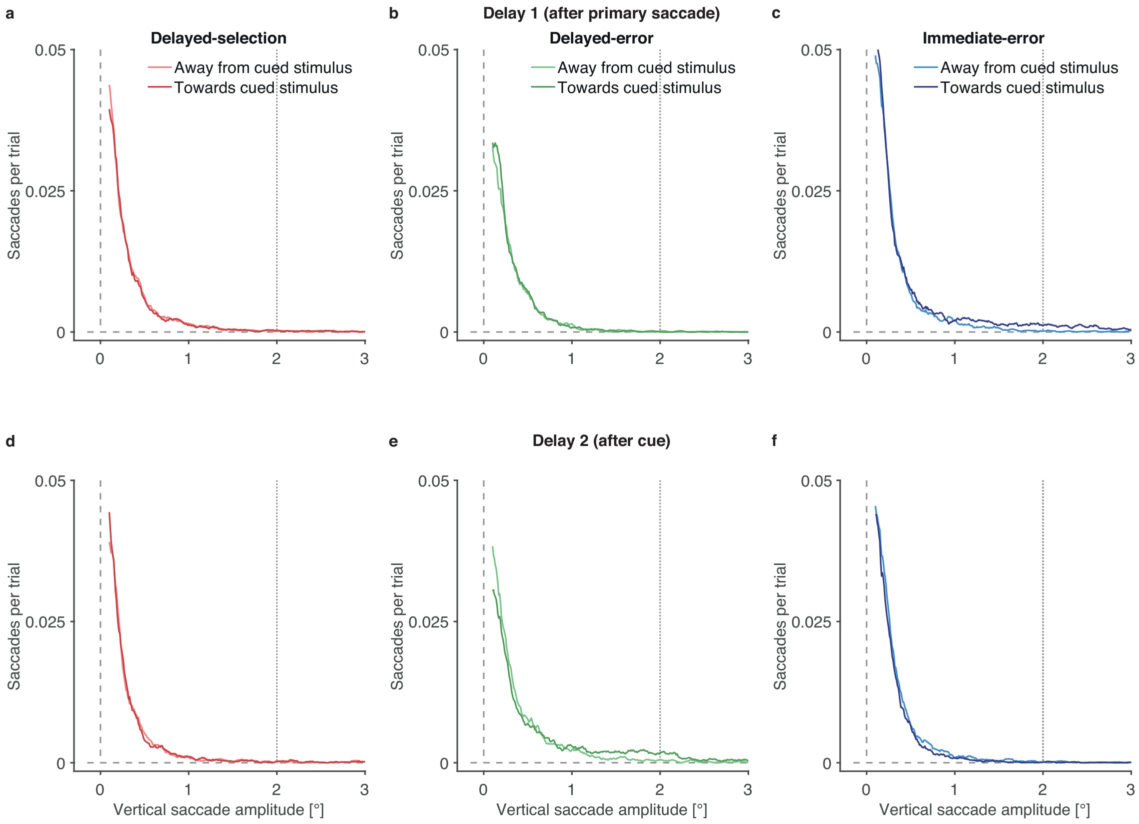

Supplementary figure S4. Saccade amplitude distribution in the saccade-only experiment. *Distribution of vertical saccade amplitudes towards (saturated colors) and away (faint colors) from the location of the task-relevant stimulus in the three conditions of the saccade-only experiment.* ***(a-c)*** *Interval between offset of the primary saccade and auditory retro-cue onset.* ***(d-f)*** *Interval between auditory retro-cue onset and reference onset. Solid colored lines indicate the number of saccades of a given amplitude, executed per trial. The grey dotted line marks the eccentricity of the stimuli.*

Supplementary References

1. Engbert, R. & Kliegl, R. Microsaccades uncover the orientation of covert attention. *Vision Res.* **43**, 1035–1045 (2003).
2. White, O. & Diedrichsen, J. Responsibility assignment in redundant systems. *Curr. Biol.* **20**, 1290–1295 (2010).
3. Spivey, M. J. & Geng, J. J. Oculomotor mechanisms activated by imagery and memory: Eye movements to absent objects. *Psychol. Res.* **65**, 235–241 (2001).
